# A Novel Small-Molecule GRP94 Modulator Increases PCSK9 Secretion and Promotes LDLR Degradation

**DOI:** 10.3390/life15081321

**Published:** 2025-08-20

**Authors:** Wenjing Yan, Yongwang Zhong, Shengyun Fang

**Affiliations:** 1 Department of Pharmacology and Physiology, University of Maryland School of Medicine, Baltimore, MD 21201, USA; yongwangzhong@som.umaryland.edu (Y.Z.); sfang@som.umaryland.edu (S.F.); 2 Program in Oncology, UM Greenebaum Comprehensive Cancer Center, University of Maryland School of Medicine, Baltimore, MD 21201, USA

**Keywords:** GRP94, PCSK9-LDLR, ER-associated degradation (ERAD), protein quality control

## Abstract

The endoplasmic reticulum (ER) maintains protein homeostasis through chaperone-mediated folding and ER-associated degradation (ERAD). Disruption of this quality control, particularly involving the ER chaperone GRP94, contributes to diseases such as hypercholesterolemia, cancer, and immune disorders, where defective GRP94-dependent folding and the trafficking of client proteins like PCSK9, integrins, and Toll-like receptors drive pathology. Here, we characterize NSC637153 (cp153), a small molecule identified in a drGFP-based ERAD dislocation screen, as a selective probe of GRP94-dependent processes. cp153 inhibits the dislocation of ERAD substrates, preferentially affecting luminal clients, increases PCSK9 secretion, and promotes LDLR degradation. Unlike ATP-competitive HSP90 inhibitors, cp153 does not induce HSP70 or destabilize AKT, suggesting that it perturbs GRP94 function by interfering with client interaction or folding. The identification of cp153 provides a useful tool to for probing GRP94’s role in protein folding, trafficking, ER quality control, and disease-relevant signaling pathways, and supports the development of client-selective GRP94-targeted therapies.

## 1. Introduction

The endoplasmic reticulum (ER) maintains proteostasis through a coordinated network of chaperones and degradation pathways that govern protein folding, quality control, and secretion. A key node in this system is the ER-associated degradation (ERAD) pathway, which enables the retrotranslocation, also known as dislocation, of misfolded proteins for proteasomal clearance [1,2,3,4,5,6]. The pharmacological modulation of ERAD has emerged as a strategy for probing secretory pathway dynamics and identifying novel therapeutic targets.

GRP94 (glucose-regulated protein 94), the ER-resident paralog of HSP90, is a selective chaperone involved in the maturation of a restricted subset of secretory and membrane-bound proteins [7]. Among its well-characterized clients is proprotein convertase subtilisin/kexin type 9 (PCSK9), which regulates cholesterol metabolism by promoting the lysosomal degradation of the LDL receptor (LDLR). This axis has drawn considerable attention in cardiovascular research, as suppressing PCSK9 secretion leads to increased LDLR surface expression and enhanced hepatic LDL clearance [8,9,10]. GRP94 also supports cancer-related processes by stabilizing proteins involved in cell migration and survival, broadening its relevance beyond lipid metabolism [11,12].

Small-molecule inhibitors have demonstrated that GRP94 can be selectively targeted [8,13,14,15,16,17,18,19,20,21,22]. Most inhibitors act by binding the N-terminal ATP-binding domain, disrupting GRP94’s chaperone cycle [8,13,14,15,16,17,18,19]. However, emerging evidence also supports alternative mechanisms, including interference with client protein engagement via allosteric or substrate-binding surfaces [20,21,22]. The functional complexity of GRP94’s client interactions and the limitations of broader HSP90 inhibitors, such as 17AAG, highlight the need for more selective chemical tools to dissect GRP94 biology.

Here, we present cp153, a novel small-molecule dislocation inhibitor identified through a drGFP-based ERAD screen as a tool to probe GRP94 function. cp153 engages GRP94 and modulates client-specific processes through a mechanism distinct from ATP-competitive HSP90 inhibitors. Its effects on PCSK9/LDLR trafficking, melanogenesis, and cell migration highlight its utility for dissecting GRP94-dependent biology.

## 2. Materials and Methods

The Cell culture

HeLa cells (ATCC^®^ CCL-2™) were cultured in Dulbecco’s Modified Eagle Medium (DMEM) supplemented with 10% heat-inactivated fetal bovine serum (FBS) and 1% penicillin–streptomycin (100 U/mL penicillin and 100 μg/mL streptomycin). Cells were grown in a humidified incubator at 37 °C with a 5% CO_2_ atmosphere. For specialized experiments, we utilized previously established stable cell lines: HeLa cells expressing SP-S11-NHK-HA or SP-S11-CD3δ-HA [23]. These engineered cell lines were maintained under identical culture conditions to the parental HeLa cells, with the addition of appropriate selection antibiotics to maintain transgene expression. All cell lines were routinely monitored for morphology and growth characteristics and were confirmed to be free of mycoplasma contamination through periodic testing.

Reagents and antibodies

All antibodies used in this study were obtained from commercial sources with the following specifications: Anti-PCSK9 (clone F-8, sc-515082) and anti-GRP94 (H-10, sc-393402) were obtained from Santa Cruz Biotechnology (Dallas, TX, USA). Anti-HSP70 (clone N15F2-5, ADI-SPA-8133) was from Enzo Biochem (New York, NY, USA). Other antibodies were used as previously described [23]. The small-molecule inhibitor cp153 was provided by the National Institutes of Health (NIH, Bethesda, MD, USA). All other chemicals and laboratory reagents, unless otherwise specified, were purchased from Millipore Sigma (St. Louis, MO, USA) and were of molecular biology or cell culture grade.

Cell Viability Assessment via WST Assay

Cell viability was quantitatively assessed using the WST colorimetric assay. Briefly, HeLa cells were seeded at a density of 3 × 10^3^ cells/well in 96-well plates and allowed to adhere overnight in complete growth medium. Cells were then treated with varying concentrations of cp153 prepared in fresh culture medium and incubated for 42 h at 37 °C under standard culture conditions. Following treatment, 10 μL of WST-8 reagent was added to each well and plates were incubated for an additional 1 h at 37 °C. Metabolic activity was quantified by measuring absorbance at 450 nm using a Bio-Rad Model 680 microplate reader.

drGFP measurement

The real-time monitoring of protein dislocation was performed using the drGFP reporter system in HeLa cells stably co-expressing SP-S11-NHK-HA and cytosolic S1-10 (NHK-drGFP) [23,24]. Cells were seeded at 2 × 10^4^ cells/well in 96-well plates and cultured overnight in complete DMEM to reach 70–80% confluence. Prior to imaging, medium was replaced with fresh serum-containing DMEM supplemented with cp153, in the presence or absence of proteasome inhibitor bortezomib (BTZ, 2 μM). GFP fluorescence was subsequently monitored every 1 h using an IncuCyte S3 live-cell analysis system equipped with a 20× objective, maintaining standard culture conditions (37 °C, 5% CO_2_) throughout the imaging period. This experimental setup allowed for the quantitative assessment of cp153’s effects on ER-to-cytosol protein dislocation in real time while controlling for potential cytotoxicity through concurrent phase-contrast imaging.

Protein degradation analysis via cycloheximide chase assay

To assess the effects of cp153 on ERAD substrate stability, we performed cycloheximide chase experiments in HeLa cells stably expressing either NHK-HA (luminal substrate) or CD3δ-HA (transmembrane substrate). Cells were seeded at 2 × 10^5^ cells/well in 12-well plates and allowed to adhere overnight under standard culture conditions. Following pretreatment with cp153 or vehicle control for 1 h, protein synthesis was halted by adding cycloheximide (100 μg/mL). Cells were harvested at defined time points, lysed in RIPA buffer supplemented with protease inhibitors, and clarified via centrifugation. Protein extracts were resolved via SDS-PAGE (10% gels) and analyzed via immunoblotting using anti-HA antibodies.

Target engagement analysis via Cellular Thermal Shift Assay (CETSA)

To investigate potential interactions between cp153 and GRP94, we performed CETSA according to established protocols with modifications [25,26,27,28]. HeLa cells were treated with cp153 or vehicle control (DMSO) for 1 h under standard culture conditions. Cells were then harvested, washed with PBS, and lysed in hypotonic buffer (25 mM Tris-HCl pH 7.5, 150 mM NaCl, and 1% NP-40) supplemented with protease inhibitors at 4 °C. The lysate was aliquoted and subjected to a temperature gradient (37–67 °C) for 3 min, followed by 3 min at 25 °C for temperature equilibration. After cooling to 4 °C, samples were centrifuged at 20,000× *g* for 20 min at 4 °C to separate soluble proteins from aggregates. Soluble fractions were analyzed via SDS-PAGE (30 μg protein/lane) and immunoblotting using antibodies against GRP94 and other ERAD components. Thermal stabilization curves were generated by quantifying band intensities normalized to the 37 °C control.

Quantification of melanin content

To evaluate the effects of cp153 on melanogenesis, we measured intracellular melanin levels in B16F10 melanoma cells following established protocols with modifications [29]. Cells were treated with cp153 or reference compounds (PU-WS13) for 40 h under standard culture conditions. After treatment, cells were washed twice with PBS to remove extracellular melanin, then lysed in 1 M of NaOH (1 mL/well) and heated at 80 °C for 15 min to solubilize melanin. Cell debris was pelleted via centrifugation (10,000× *g*, 5 min), and the absorbance of supernatants was measured at 490 nm using a spectrophotometer. The melanin content was normalized to total cellular protein determined via BCA assay and expressed as a percentage relative to vehicle-treated controls.

Protein analysis via Western blotting

For Western blot analysis, cells treated with experimental compounds were harvested and lysed in ice-cold RIPA buffer supplemented with protease and phosphatase inhibitor cocktails. Lysates were clarified via centrifugation at 12,000× *g* for 15 min at 4 °C, and protein concentrations were determined using the Bradford assay with BSA standards. Equal amounts of protein (30 μg per lane) were separated via electrophoresis on 11% SDS-polyacrylamide gels and transferred to PVDF membranes using a semi-dry transfer system. Membranes were blocked with 5% non-fat dry milk in TBST (Tris-buffered saline with 0.1% Tween-20) for 1 h at room temperature with gentle agitation. Primary antibody incubations were performed overnight at 4 °C. After three 10 min TBST washes, membranes were incubated with species-matched HRP-conjugated secondary antibodies (1:5000 in 5% milk) for 1 h at room temperature. Following additional washes, immunoreactive bands were detected using SuperSignal West Pico PLUS or Femto Maximum Sensitivity chemiluminescent substrates.

Cell migration assessment via wound healing assay

To evaluate the anti-migratory effects of cp153, we performed standardized wound healing assays in HeLa cells. Cells were seeded in 24-well plates and cultured until forming a confluent monolayer. Using a sterile 10 μL pipette tip, two parallel linear wounds were created per well, followed by two gentle PBS washes to remove detached cells. Immediately after wounding (0 h baseline), brightfield images were captured at marked positions. Cells were then treated with cp153. Wound closure was monitored under standard culture conditions (37 °C, 5% CO_2_), with image acquisition at each time point maintaining identical focal positions. Migration rates were quantified by measuring wound area reduction using ImageJ (1.52a) software (National Institutes of Health, Bethesda, MD, USA).

Statistical analysis

The data were expressed as the mean ± SD of the results obtained from at least three independent experiments. Differences between two groups were analyzed using an unpaired two-tailed Student’s *t*-test using GraphPad Prism 7.0. For all analyses, the *p*-value was considered significant as follows: * *p*  <  0.05, ** *p*  <  0.01. Data visualization was performed using GraphPad Prism. Normality was assumed based on the consistency of biological replicates and previous experience with similar datasets; however, no formal normality testing was performed in this study. In future work, we will conduct normality assessments (e.g., the Shapiro–Wilk test) before selecting statistical tests, applying nonparametric alternatives when appropriate.

## 3. Results

### 3.1. cp153 Inhibits ER-Associated Protein Dislocation and Substrate Ubiquitination

**Figure 1:**
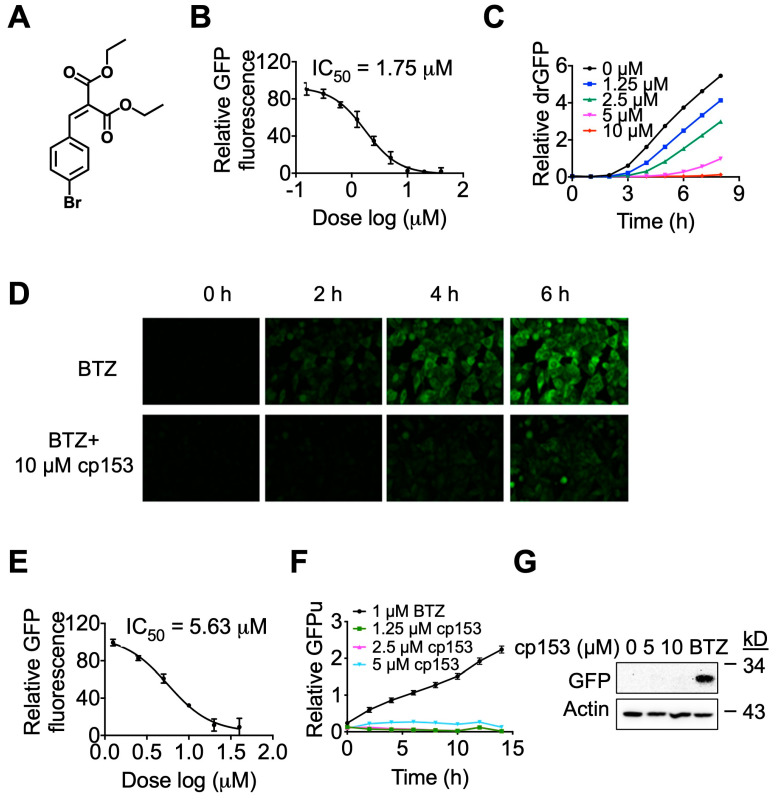
cp153 is a dislocation inhibitor. (**A**) Chemical structure of cp153, a small-molecule inhibitor identified through drGFP-based screening. (**B**) Dose–response curve showing cp153 inhibition of NHK dislocation (IC_50_ = 1.75 μM) in the drGFP assay. HeLa cells expressing NHK-drGFP were treated with cp153 and BTZ for 6 h. (**C**,**D**) Time-lapse imaging of drGFP fluorescence in HeLa cells expressing the ERAD substrate NHK. (**C**) Quantification of drGFP fluorescence intensity. (**D**) Representative fluorescence images from (**C**). Treatment with the proteasome inhibitor BTZ alone induced progressive GFP reconstitution due to NHK dislocation (top panel), while co-treatment with cp153 (10 µM) nearly abolished this signal (bottom panel), demonstrating potent inhibition of dislocation. (**E**) cp153 inhibition of CD3δ dislocation (IC_50_ = 5.63 μM) in the drGFP assay (conditions as in B). (**F**,**G**) GFPu reporter assay showing that cp153 does not accumulate GFPu like BTZ (**F**). Western blot confirmation of GFPu levels. β-actin: loading control (**G**).

In our previous studies [23,30], we employed a live-cell reporter assay, drGFP, to screen for small-molecule modulators of dislocation, also known as retrotranslocation, during ERAD and identified a small molecule NSC637153 (diethyl (4-bromobenzylidene) malonate), cp153 in short, as a novel dislocation inhibitor (Figure 1A). In this study, we further characterized cp153’s dislocation inhibitory activity. cp153 inhibited the dislocation of the null Hong Kong variant of α-1-antitrypsin (NHK), a well-characterized luminal ERAD substrate with an IC_50_ of 1.75 μM (Figure 1B). The inhibitory activity was confirmed via the time-lapse imaging of cells expressing SP-S11-NHK-HA (Figure 1C). While treatment with the proteasome inhibitor bortezomib (BTZ) alone resulted in the time-dependent accumulation of GFP fluorescence (reflecting the dislocation of the substrate), co-treatment with cp153 nearly abolished this signal (Figure 1D), indicating the effective blockade of protein dislocation.

To assess whether cp153’s activity extended beyond luminal substrates, we tested its effects on CD3δ, a single-pass transmembrane ERAD substrate. cp153 similarly inhibited CD3δ dislocation, though with slightly reduced potency (IC_50_ = 5.63 μM; Figure 1E). These results collectively suggest cp153 as a broad-spectrum dislocation inhibitor capable of blocking the ERAD of both luminal and transmembrane substrates.

To distinguish cp153’s mechanism from proteasome inhibitors, we tested its effects on GFPu, a cytosolic proteasome reporter. While BTZ caused robust GFPu accumulation as expected, cp153 showed no effect (Figure 1F,G), confirming that it does not impair general proteasome function. This selectivity contrasts with classical ERAD inhibitors that act at later degradation steps, positioning cp153 as an early-step dislocation blocker.

### 3.2. cp153 Inhibits Ubiquitination and Stabilizes ERAD Substrates While Inducing ER Stress

**Figure 2:**
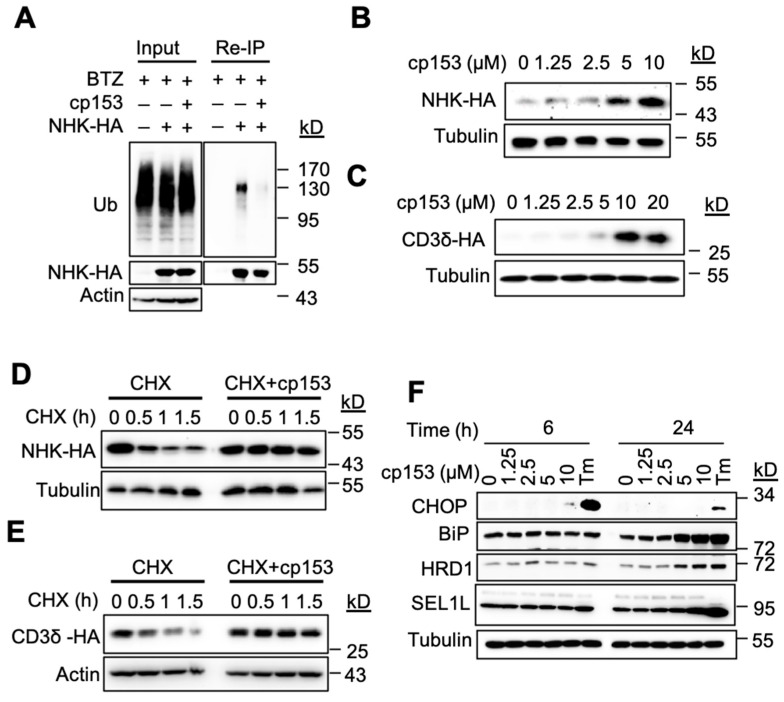
cp153 inhibits ERAD and induces ER stress. (**A**) cp153 reduces NHK ubiquitination. Re-immunoprecipitation (Re-IP) of HA-tagged NHK from HeLa cells treated with cp153 or DMSO for 4 h in the presence of BTZ. Immunoblots show ubiquitin (Ub) and NHK-HA levels. Input: 5% of total lysate. (**B**,**C**) cp153 stabilized NHK (**B**) and CD3δ (**C**) in a dose-dependent manner. 293 cells stably expressing NHK-HA or CD3δ-HA were treated with cp153 overnight and then processed for IB. (**D**,**E**) NHK (**D**) or CD3δ (**E**) degradation was determined via cycloheximide (CHX) chase. (**F**) HeLa cells were treated with increasing concentrations of cp153 for 24 h and processed for immunoblotting. Tunicamycin (Tm) was used as a positive control.

Protein dislocation is a prerequisite for the ubiquitination and degradation of ER luminal substrates [1,31]. To characterize cp153’s effects on this process, we examined its impact on the model ERAD substrate NHK. cp153 treatment significantly reduced NHK ubiquitination compared to control cells (Figure 2A). To further characterize cp153’s effects on ERAD substrates, we analyzed HA-tagged NHK and CD3δ levels in stably expressing cell lines. cp153 treatment led to the dose-dependent stabilization of both substrates (Figure 2B,C), consistent with its dislocation-inhibiting activity. Cycloheximide chase experiments revealed that cp153 (10 μM) nearly completely blocked the degradation of both NHK (Figure 2D) and CD3δ (Figure 2E), confirming its broad inhibition of ERAD-mediated protein turnover. The similar stabilization patterns observed for these structurally distinct substrates suggest that cp153 targets a common, early step in the dislocation process. As the inhibition of ERAD typically leads to the accumulation of misfolded proteins in the ER lumen, we examined whether cp153 treatment induced ER stress. Our analysis revealed that cp153 treatment specifically upregulated key components of the ER quality control system. Western blot analysis demonstrated dose-dependent increases in the ERAD ubiquitin ligase Hrd1 and the major ER chaperone BiP, with the corresponding induction of the pro-apoptotic transcription factor CHOP (Figure 2F). These changes were observed following 6 h and 24 h treatment with cp153 (0–10 μM), suggesting the activation of both adaptive (BiP) and terminal (CHOP) unfolded protein response pathways. The response pattern mirrored that of tunicamycin (Tm)-treated positive controls.

### 3.3. GRP94 Is a Potential Target of cp153

**Figure 3:**
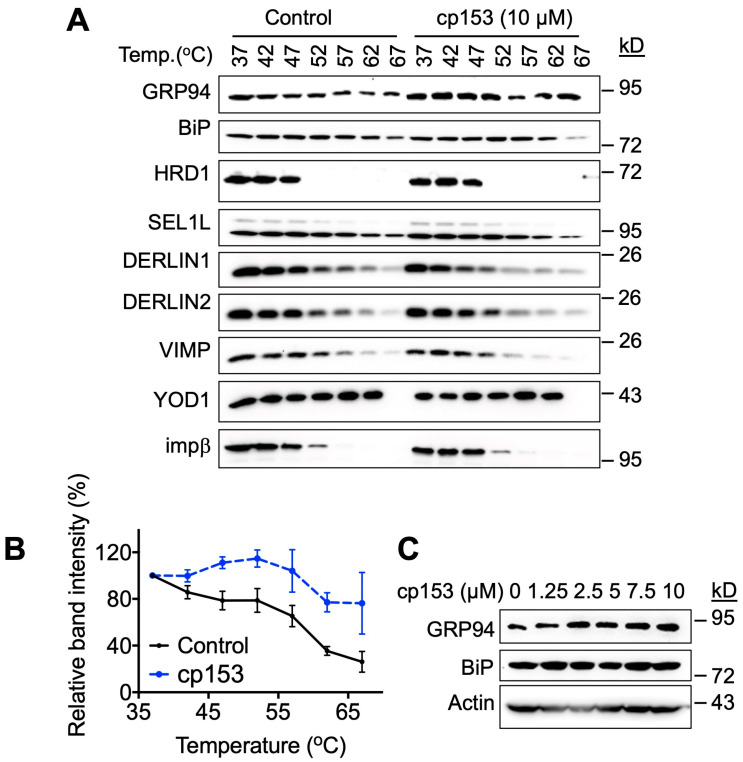
GRP94 is a potential target of cp153. (**A**) Thermal shift assay of HeLa cell lysates treated with 10 μM cp153 or DMSO control. Immunoblots show soluble fractions of GRP94 and other proteins after heating to indicated temperatures (37–67 °C). (**B**) Quantification of thermal stabilization profiles for GRP94 from panel A. Data represent band intensity normalized to 37 °C control (mean ± SD, *n* = 3). (**C**) Dose-dependent thermal stabilization of GRP94 at 67 °C, showing increased soluble GRP94 levels with cp153 treatment (0–10 μM).

To investigate cp153’s mechanism of action, we employed the Cellular Thermal Shift Assay (CETSA) to screen for potential target engagement among ERAD-related proteins. This approach exploits the principle of the ligand-induced thermal stabilization of target proteins [25,26,27,28]. Initial screening across a temperature gradient (37–67 °C) revealed a significant thermal shift, specifically for GRP94 following 1 h treatment with 10 µM of cp153 (Figure 3A,B), while the other ERAD components examined showed no effect. Based on the thermal denaturation profile of GRP94, we selected 67 °C for subsequent dose–response experiments. Consistent with target engagement, cp153 treatment resulted in the dose-dependent stabilization of GRP94 (Figure 3C), with increased protein levels detectable at temperatures that denatured GRP94 in untreated controls. This concentration-dependent thermal stabilization strongly suggests a physical interaction between cp153 and GRP94. These results suggest that GRP94 is a cellular target of cp153 in the ERAD pathway.

### 3.4. cp153 Modulates PCSK9 Secretion and LDLR Degradation Without Inducing Cytosolic HSP90 Responses

**Figure 4:**
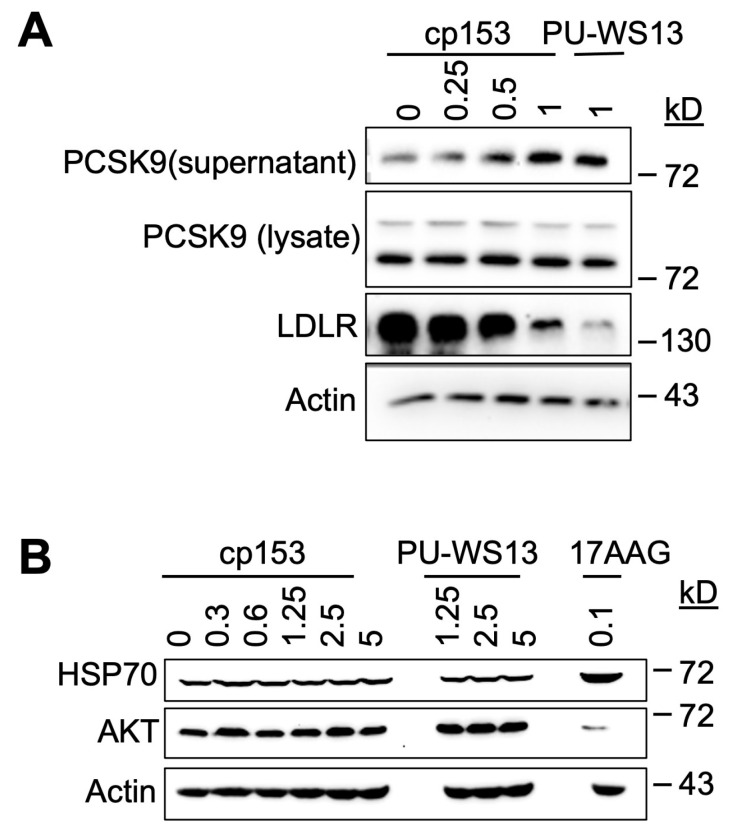
cp153 increases PCSK9 secretion and LDLR degradation without inducing cytosolic HSP90 responses. (**A**) Immunoblots of PCSK9 (supernatant/lysate) and LDLR in cells treated with cp153 or PU-WS13 for 24 h. (**B**) HSP70 and AKT levels after treatments with cp153, PU-WS13, or 17AAG. β-actin: loading control.

GRP94 is a key ER chaperone responsible for the proper folding and secretion of specific client proteins, including PCSK9, which in turn regulates LDL receptor (LDLR) turnover [8,32]. To determine whether cp153 interferes with this GRP94-dependent pathway, we examined its impact on PCSK9 secretion and LDLR expression in cultured cells. cp153 treatment led to a significant increase in secreted PCSK9 levels, accompanied by a reduction in LDLR abundance (Figure 4A). Notably, this phenotype was mirrored by PU-WS13, a selective GRP94 inhibitor known to bind outside the ATP pocket and disrupt client protein interaction 13–14. These shared effects suggest that cp153 may similarly impair GRP94’s client-handling capacity, possibly at a site affecting client release, substrate dwell time, or ER exit, rather than inhibiting ATP hydrolysis directly.

The total intracellular PCSK9 levels remained unchanged upon cp153 treatment, indicating that the compound does not impact PCSK9 synthesis, but instead modulates its trafficking or release from the ER. This pattern is consistent with selective interference in post-translational chaperone function [33,34,35].

To assess cp153’s specificity, we evaluated two classical markers of cytosolic Hsp90 inhibition—HSP70 induction and AKT destabilization—which are typically observed with pan-Hsp90 inhibitors such as 17AAG. As expected, 17AAG treatment triggered robust HSP70 upregulation [36,37] and reduced AKT protein levels [38,39], consistent with the global disruption of HSP90 ATPase activity. In contrast, cp153 had no effect on either marker (Figure 4B), indicating that it does not significantly inhibit cytosolic HSP90 isoforms or induce a generalized heat shock response.

These findings point to a selective mechanism by which cp153 perturbs GRP94-mediated client maturation, resulting in altered PCSK9–LDLR dynamics. The similarity between cp153 and PU-WS13 supports the notion that cp153 may target a noncanonical regulatory region in GRP94, potentially affecting client recognition or trafficking, rather than competing at the ATP-binding site as 17AAG does. This selective activity underscores cp153’s utility as a chemical probe in dissecting GRP94-dependent secretory functions without engaging the broader Hsp90 network.

### 3.5. cp153 Inhibits HeLa Cell Migration and Melanin Production

**Figure 5:**
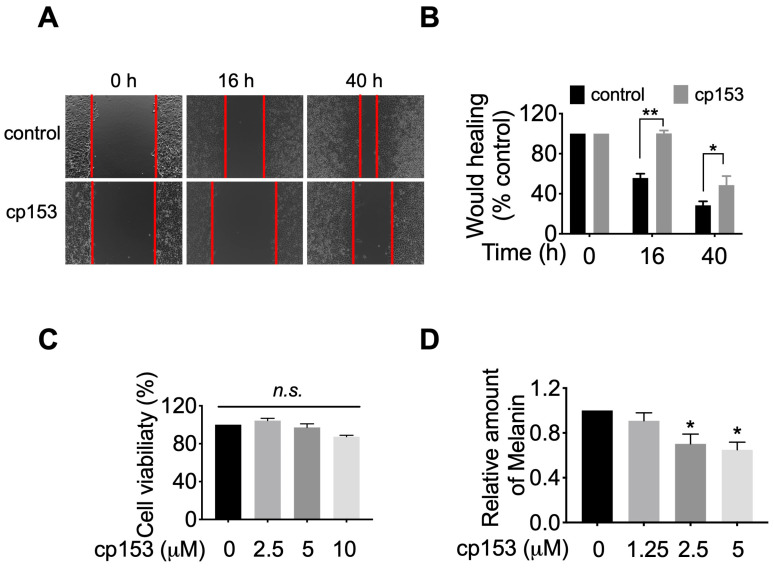
cp153 inhibits HeLa cell migration and melanin production. (**A**) Representative wound images (0, 16, and 40 h) with cp153 or DMSO control. (**B**) Quantified wound closure (mean ± SD, *n* = 3). (**C**) Cell viability after 40 h treatment. (**D**) Melanin content in B16F10 cells after 40 h treatment, with indicated concentrations of cp153 or PU-WS13. Data normalized to DMSO control (mean ± SD, *n* = 3). Unpaired two-tailed Student’s *t*-test was performed. * *p* <  0.05, ** *p* <  0.01, and n.s. means not significant.

Given GRP94’s established role in maturing integrins and other motility-related client proteins [8,40,41], we hypothesized that cp153-mediated GRP94 inhibition would impair cancer cell migration. To test this, we performed wound healing assays in HeLa cells, which robustly express GRP94-dependent adhesion machinery. Brightfield microscopy images captured at 0 h, 16 h, and 40 h post scratching revealed that cp153 treatment significantly impaired wound closure compared to controls (Figure 5A). Quantitative analysis demonstrated that cp153 significantly inhibited cell migration, with cp153 reducing wound closure by ~70% relative to DMSO-treated cells after 40 h (Figure 5B). Importantly, cp153 treatment did not affect cell viability at any concentration tested (Figure 5C), confirming that the observed anti-migratory effects were not due to cytotoxicity. These findings align with the established mechanisms of GRP94 inhibition, where impaired chaperone function leads to the reduced cell surface expression of integrins and other motility-related client proteins.

Since GRP94 is required for the maturation and trafficking of tyrosinase [42,43], the rate-limiting enzyme in melanin synthesis, we hypothesized that cp153-mediated GRP94 inhibition would impair melanogenesis. To test this, we examined cp153’s effects on melanin production in B16F10 murine melanoma cells, a well-established model for studying pigment biosynthesis. Quantitative analysis revealed that cp153 treatment (1.25–5 μM) for 40 h reduced melanin production in B16F10 cells (Figure 5D). This dose-dependent suppression of melanogenesis suggests that cp153 interferes with the GRP94-dependent processing of melanogenic enzymes, consistent with its proposed mechanism as a selective GRP94 modulator.

## 4. Discussion

Building on our ongoing efforts to identify small-molecule modulators of ER-associated degradation (ERAD) [23,30,44,45,46], we discovered cp153 through the systematic screening of a chemical library, using our established drGFP-based dislocation reporter system. cp153 consistently inhibited protein dislocation across multiple validation assays (Figure 1 and Figure 2), demonstrating potent activity against both luminal (NHK) and transmembrane (CD3δ) ERAD substrates. Its preferential inhibition of the luminal substrate NHK suggests the selective engagement of luminal dislocation pathways, potentially involving ER-resident chaperones or components of the SEL1L–HRD1 complex.

Among the possible molecular targets, multiple lines of evidence point to GRP94 as a candidate mediator of cp153’s effects. GRP94, an ER-resident HSP90 paralog, governs the folding and trafficking of secretory and membrane proteins, including several implicated in cancer and metabolic regulation [7,12,18]. The CETSA results showed the thermal stabilization of GRP94 in the presence of cp153 (Figure 3), suggesting GRP94 as its target. Although CETSA-based thermal stabilization supports the possibility that cp153 engages GRP94, the absence of direct binding evidence—such as from ITC, SPR, or affinity pull-down assays—remains a limitation of our study. Future investigations employing biophysical and chemoproteomic approaches will be essential to confirm this interaction and define the precise binding site.

GRP94 engagement is further supported by cp153’s biological activities on a known client protein for GRP94, PCSK9. cp153 increases PCSK9 secretion and reduces LDLR abundance (Figure 4A). In the PCSK9–LDLR axis, a pathway tightly regulated by GRP94 chaperone function, canonical ATP-binding inhibitors typically reduce PCSK9 secretion, resulting in increased LDLR levels on the cell surface [8]. In contrast, cp153 markedly increased PCSK9 secretion and decreased LDLR abundance, an effect also observed with PU-WS13, a non-ATP-competitive GRP94 inhibitor. This paradoxical phenotype may reflect a unique mechanism through which cp153 modulates chaperone–client dynamics, possibly by shortening the residence time on GRP94 or interfering with ER quality control checkpoints, thereby enhancing PCSK9 release and trafficking without impairing folding. Further studies are needed to determine whether cp153 affects substrate trafficking or ER exit in a manner similar to PU-WS13.

The fact that cp153 does not induce cytosolic stress responses such as HSP70 upregulation or AKT destabilization (Figure 4B) further supports a selective mode of action, possibly through allosteric interaction or substrate interface disruption rather than global inhibition. Our study does not exclude the possibility of cp153 interacting with other HSP90 paralogs. However, the absence of HSP70 induction and AKT destabilization—two well-established readouts of cytosolic HSP90 inhibition—suggests that cp153’s effects are more specific to GRP94. Future CETSA-based profiling or genetic rescue experiments will be valuable for fully assessing selectivity.

While our study focused on the dislocation of GRP94 client proteins, we did not directly examine the fate of lectin-based ER chaperones such as calnexin or calreticulin. Given cp153’s ability to induce ER stress and affect protein trafficking, future studies will be needed to assess whether lectin chaperone function or expression is altered, potentially contributing to the observed phenotypes.

This hypothesis is further supported by cp153’s broader phenotypic effects, which mirror known GRP94 functions. Beyond ERAD inhibition, cp153 suppressed melanogenesis and inhibited HeLa cell migration (Figure 5)—two processes linked to GRP94’s role in folding metastasis-promoting proteins such as integrins [8,40,41], matrix metalloproteinases (MMPs) [7,42], and melanogenic regulators like tyrosinase [42,43] and MITF [47]. cp153 treatment led to a dose-dependent reduction in melanin production in B16F10 cells. While this observation is consistent with potential interference in GRP94-dependent melanogenesis pathways, such as tyrosinase folding or trafficking, we did not assess tyrosinase expression or activity directly. Further studies will be required to determine whether cp153 modulates specific melanogenic enzymes or regulatory factors.

Although our findings tentatively implicate GRP94 in cp153’s mechanism of action, further studies are required to confirm direct target engagement and define the precise mode of inhibition. Structural analyses, chemoproteomics, and genetic approaches will be crucial for validating target specificity and distinguishing between direct interaction and downstream pathway modulation. Although cp153 is unlikely to serve as a cholesterol-lowering agent, its ability to selectively modulate GRP94 function—without inducing cytosolic HSP90 stress responses—makes it a promising tool for modeling diseases where secretory pathway dysregulation is central. These may include cancers involving integrin trafficking, autoimmune conditions linked to Toll-like receptor signaling, and pigmentary diseases dependent on tyrosinase maturation.

## 5. Conclusions

In summary, cp153 emerges as a promising chemical probe for dissecting ERAD dynamics and GRP94-dependent client maturation. While its effects on PCSK9 secretion and LDLR degradation preclude its immediate application as a cholesterol-lowering agent, cp153 offers unique value for studying chaperone–client biology within the secretory pathway. Its apparent selectivity for luminal dislocation and its functional divergence from ATP-competitive inhibitors highlight the potential of targeting chaperone–substrate interactions as a more nuanced strategy in chemical biology and therapeutic development.

## Data Availability

The original contributions presented in this study are included in the article. Further inquiries can be directed to the corresponding author.

## Abbreviations

The following abbreviations are used in this manuscript:

ER	Endoplasmic reticulum
ERAD	ER-associated degradation
CETSA	Cellular Thermal Shift Assay

## References

[1] Vembar S.S., Brodsky J.L. (2008). One step at a time: Endoplasmic reticulum-associated degradation. Nat. Rev. Mol. Cell Biol..

[2] Christianson J.C., Jarosch E., Sommer T. (2023). Mechanisms of substrate processing during ER-associated protein degradation. Nat. Rev. Mol. Cell Biol..

[3] Wu S.A., Li Z.J., Qi L. (2025). Endoplasmic reticulum (ER) protein degradation by ER-associated degradation and ER-phagy. Trends Cell Biol..

[4] Hebert D.N., Molinari M. (2007). In and out of the ER: Protein folding, quality control, degradation, and related human diseases. Physiol. Rev..

[5] Tsai B., Ye Y., Rapoport T.A. (2002). Retro-translocation of proteins from the endoplasmic reticulum into the cytosol. Nat. Rev. Mol. Cell Biol..

[6] Liu Y., Ye Y. (2011). Proteostasis regulation at the endoplasmic reticulum: A new perturbation site for targeted cancer therapy. Cell Res..

[7] Wu B.X., Hong F., Zhang Y., Ansa-Addo E., Li Z. (2016). GRP94/gp96 in Cancer: Biology, Structure, Immunology, and Drug Development. Adv. Cancer Res..

[8] Poirier S., Mamarbachi M., Chen W.T., Lee A.S., Mayer G. (2015). GRP94 Regulates Circulating Cholesterol Levels Through Blockade of PCSK9-Induced LDLR Degradation. Cell Rep..

[9] Guo S., Xia X.D., Gu H.M., Zhang D.W. (2020). Proprotein Convertase Subtilisin/Kexin-Type 9 and Lipid Metabolism. Adv. Exp. Med. Biol..

[10] Lebeau P.F., Platko K., Byun J.H., Makda Y., Austin R.C. (2022). The Emerging Roles of Intracellular PCSK9 and Their Implications in Endoplasmic Reticulum Stress and Metabolic Diseases. Metabolites.

[11] Lee A. (2014). Glucose-regulated proteins in cancer: Molecular mechanisms and therapeutic potential. Nat. Rev. Cancer.

[12] Duan X.F., Iwanowycz S., Ngoi S., Hill M., Zhao Q., Liu B. (2021). Molecular Chaperone GRP94/GP96 in Cancers: Oncogenesis and Therapeutic Target. Front. Oncol..

[13] Duerfeldt A.S., Peterson L.B., Maynard J.C., Ng C.L., Eletto D., Ostrovsky O., Shinogle H.E., Moore D.S., Argon Y., Nicchitta C.V. (2012). Development of a Grp94 inhibitor. J. Am. Chem. Soc..

[14] Patel P.D., Yan P., Seidler P.M., Patel H.J., Sun W., Yang C., Que N.S., Taldone T., Finotti P., Stephani R.A. (2013). Paralog-selective Hsp90 inhibitors define tumor-specific regulation of HER2. Nat. Chem. Biol..

[15] Immormino R.M., Metzger L.E., Reardon P.N., Dollins D.E., Blagg B.S.J., Gewirth D.T. (2009). Different poses for ligand and chaperone in inhibitor-bound Hsp90 and GRP94: Implications for paralog-specific drug design. J. Mol. Biol..

[16] Mishra S.J., Ghosh S., Stothert A.R., Dickey C.A., Blagg B.S.J. (2017). Transformation of the Non-Selective Aminocyclohexanol-Based Hsp90 Inhibitor into a Grp94-Seletive Scaffold. ACS Chem. Biol..

[17] Soldano K.L., Jivan A., Nicchitta C.V., Gewirth D.T. (2003). Structure of the N-terminal domain of GRP94. Basis for ligand specificity and regulation. J. Biol. Chem..

[18] Kim J.W., Cho Y.B., Lee S. (2021). Cell Surface GRP94 as a Novel Emerging Therapeutic Target for Monoclonal Antibody Cancer Therapy. Cells.

[19] Pugh K.W., Alnaed M., Brackett C.M., Blagg B.S.J. (2022). The biology and inhibition of glucose-regulated protein 94/gp96. Med. Res. Rev..

[20] Amankwah Y.S., Fleifil Y., Unruh E., Collins P., Wang Y., Vitou K., Bates A., Obaseki I., Sugoor M., Alao J.P. (2024). Structural transitions modulate the chaperone activities of Grp94. Proc. Natl. Acad. Sci. USA.

[21] Azam T.P., Han L., Deans E.E., Huang B., Hoxie R., Friedman L.J., Gelles J., Street T.O. (2025). Mechanism of client loading from BiP to Grp94 and its disruption by select inhibitors. Nat. Commun..

[22] Que N.L.S., Crowley V.M., Duerfeldt A.S., Zhao J., Kent C.N., Blagg B.S.J., Gewirth D.T. (2018). Structure Based Design of a Grp94-Selective Inhibitor: Exploiting a Key Residue in Grp94 to Optimize Paralog-Selective Binding. J. Med. Chem..

[23] Zhong Y.W., Fang S.Y. (2012). Live cell imaging of protein dislocation from the endoplasmic reticulum. J. Biol. Chem..

[24] Zhong Y.W., Shen H., Wang Y., Yang Y., Yang P., Fang S.Y. (2015). Identification of ERAD components essential for dislocation of the null Hong Kong variant of α-1-antitrypsin (NHK). Biochem. Biophys. Res. Commun..

[25] Martinez Molina D., Jafari R., Ignatushchenko M., Seki T., Larsson E.A., Dan C., Sreekumar L., Cao Y., Nordlund P. (2013). Monitoring drug target engagement in cells and tissues using the cellular thermal shift assay. Science.

[26] Martinez Molina D., Nordlund P. (2016). The Cellular Thermal Shift Assay: A Novel Biophysical Assay for In Situ Drug Target Engagement and Mechanistic Biomarker Studies. Annu. Rev. Pharmacol. Toxicol..

[27] Martinez N.J., Asawa R.R., Cyr M.G., Zakharov A., Urban D.J., Roth J.S., Wallgren E., Klumpp-Thomas C., Coussens N.P., Rai G. (2018). A widely-applicable high-throughput cellular thermal shift assay (CETSA) using split Nano Luciferase. Sci. Rep..

[28] Reinhard F.B., Eberhard D., Werner T., Franken H., Childs D., Doce C., Savitski M.F., Huber W., Bantscheff M., Savitski M.M. (2015). Thermal proteome profiling monitors ligand interactions with cellular membrane proteins. Nat. Methods.

[29] Ahn J.H., Jin S.H., Kang H.Y. (2008). LPS induces melanogenesis through p38 MAPK activation in human melanocytes. Arch. Dermatol. Res..

[30] Ruan J.J., Rothan H.A., Zhong Y.W., Yan W.J., Henderson M.J., Chen F.H., Fang S.Y. (2019). A small molecule inhibitor of ER-to-cytosol protein dislocation exhibits anti-dengue and anti-Zika virus activity. Sci. Rep..

[31] Christianson J.C., Ye Y.H. (2014). Cleaning up in the endoplasmic reticulum: Ubiquitin in charge. Nat. Struct. Mol. Biol..

[32] Lebeau P., Platko K., Al-Hashimi A.A., Byun J.H., Lhoták Š., Holzapfel N., Gyulay G., Igdoura S.A., Cool D.R., Trigatti B. (2018). Loss-of-function PCSK9 mutants evade the unfolded protein response sensor GRP78 and fail to induce endoplasmic reticulum stress when retained. J. Biol. Chem..

[33] Robinson C.M., Duggan A., Forrester A. (2024). ER exit in physiology and disease. Front Mol. Biosci..

[34] Gomez-Navarro N., Maldutyte J., Poljak K., Peak-Chew S.Y., Orme J., Bisnett B.J., Lamb C.H., Boyce M., Gianni D., Miller E.A. (2022). Selective inhibition of protein secretion by abrogating receptor-coat interactions during ER export. Proc. Natl. Acad. Sci. USA.

[35] Bao X., Liang Y., Chang H., Cai T., Feng B., Gordon K., Zhu Y., Shi H., He Y., Xie L. (2024). Targeting proprotein convertase subtilisin/kexin type 9 (PCSK9): From bench to bedside. Signal Transduct. Target. Ther..

[36] Liu M., Li M., Zhou Y., Zhou Q., Jiang Y. (2020). HSP90 inhibitor 17AAG attenuates sevoflurane-induced neurotoxicity in rats and human neuroglioma cells via induction of HSP70. J. Transl. Med..

[37] Trepel J., Mollapour M., Giaccone G., Neckers L. (2010). Targeting the dynamic HSP90 complex in cancer. Nat. Rev. Cancer.

[38] Sato S., Fujita N., Tsuruo T. (2000). Modulation of Akt kinase activity by binding to Hsp90. Proc. Natl. Acad. Sci. USA.

[39] Hong L.J., Chen A.J., Li F.Z., Chen K.J., Fang S. (2020). The HSP90 Inhibitor, 17-AAG, Influences the Activation and Proliferation of T Lymphocytes via AKT/GSK3β Signaling in MRL/lpr Mice. Drug Des. Devel. Ther..

[40] Wanderling S., Simen B.B., Ostrovsky O., Ahmed N.T., Vogen S.M., Gidalevitz T., Argon Y. (2007). GRP94 is essential for mesoderm induction and muscle development because it regulates insulin-like growth factor secretion. Mol. Biol. Cell..

[41] Zhou L., Velegraki M., Wang Y., Mandula J.K., Chang Y., Liu W., Song N.J., Kwon H., Xiao T., Bolyard C. (2024). Spatial and functional targeting of intratumoral Tregs reverses CD8+ T cell exhaustion and promotes cancer immunotherapy. J. Clin. Investig..

[42] Wang X.Y., Sun X., Chen X., Facciponte J., Repasky E.A., Kane J., Subjeck J.R. (2010). Superior antitumor response induced by large stress protein chaperoned protein antigen compared with peptide antigen. J. Immunol..

[43] Bleifuss E., Bendz H., Sirch B., Thompson S., Brandl A., Milani V., Graner M.W., Drexler I., Kuppner M., Katsanis E. (2008). Differential capacity of chaperone-rich lysates in cross-presenting human endogenous and exogenous melanoma differentiation antigens. Int. J. Hyperth..

[44] Rothan H.A., Zhong Y.W., Sanborn M.A., Teoh T.C., Ruan J.J., Yusof R., Hang J., Henderson M.J., Fang S.Y. (2019). Small molecule grp94 inhibitors block dengue and Zika virus replication. Antivir. Res..

[45] Ruan J.J., Liang D.D., Yan W.J., Zhong Y.W., Talley D.C., Rai G., Tao D.Y., LeClair C.A., Simeonov A., Zhang Y.H. (2022). A small-molecule inhibitor and degrader of the RNF5 ubiquitin ligase. Mol. Biol. Cell..

[46] Yan W.J., Zhong Y.W., Hu X., Xu T., Zhang Y.H., Kales S., Qu Y., Talley D.C., Baljinnyam B., LeClair C.A. (2023). Auranofin targets UBA1 and enhances UBA1 activity by facilitating ubiquitin trans-thioesterification to E2 ubiquitin-conjugating enzymes. Nat. Commun..

[47] Chauhan J.S., Hölzel M., Lambert J.P., Buffa F.M., Goding C.R. (2022). The MITF regulatory network in melanoma. Pigment Cell Melanoma Res..

